# Chromosome-level Genome Assembly of *Theretra japonica* (Lepidoptera: Sphingidae)

**DOI:** 10.1038/s41597-024-03500-z

**Published:** 2024-07-12

**Authors:** Ming Yan, Bao-Shan Su, Yi-Xin Huang, Zhen-Bang Xu, Zhuo-Heng Jiang, Xu Wang

**Affiliations:** 1 Anhui Provincial Key Laboratory of the Conservation and Exploitation of Biological Resources, College of Life Sciences, Anhui Normal University, Wuhu, Anhui 241000 China; 2 Collaborative Innovation Center of Recovery and Reconstruction of Degraded Ecosystem in Wanjiang Basin Co-founded by Anhui Province and Ministry of Education, School of Ecology and Environment, Anhui Normal University, Wuhu, Anhui 241000 China; 3 Key Laboratory of Zoological Systematics and Evolution, Institute of Zoology, Chinese Academy of Sciences, Beijing, 100101 China; 4 Guangxi Institute of Botany, Chinses Academy of Sciences, Guilin, Guangxi 541006 China; 5 School of Life Science, Westlake University, Hangzhou, Zhejiang 310023 China

**Keywords:** Entomology, Computational biology and bioinformatics

## Abstract

*Theretra japonica* is an important pollinator and agricultural pest in the family Sphingidae with a wide range of host plants. High-quality genomic resources facilitate investigations into behavioral ecology, morphological and physiological adaptations, and the evolution of genomic architecture. However, chromosome-level genome of *T. japonica* is still lacking. Here we sequenced and assembled the high-quality genome of *T. japonica* by combining PacBio long reads, Illumina short reads, and Hi-C data. The genome was contained in 95 scaffolds with an accumulated length of 409.55 Mb (BUSCO calculated a genome completeness of 99.2%). The 29 pseudochromosomes had a combined length of 403.77 Mb, with a mapping rate of 98.59%. The genomic characterisation of *T. japonica* will contribute to further studies for Sphingidae and Lepidoptera.

## Background & Summary

Sphingidae, commonly recognized as hawkmoth, is a member of the Lepidoptera, currently boasting over 1,460 recorded species worldwide^1,2^. They are medium to large, heavy-bodied insects with bullet-shaped bodies and long, blade-like wings. Hawkmoths are known for their powerful flight, which can reach speeds of 40–50 kilometers per hour. Numerous hawkmoths are globally recognized as agricultural and forestry pests, including species such as *Clanis undulosa* Moore, 1879, *Theretra oldenlandiae* (Fabricius, 1775), and *Ampelophaga rubiginosa* Bremer & Grey, 1853, etc.^3^. The larvae of hawkmoths, which are known to inflict substantial economic harm on crops, predominantly survive by feeding on the foliage of trees and vegetables.

**Fig. 1:**
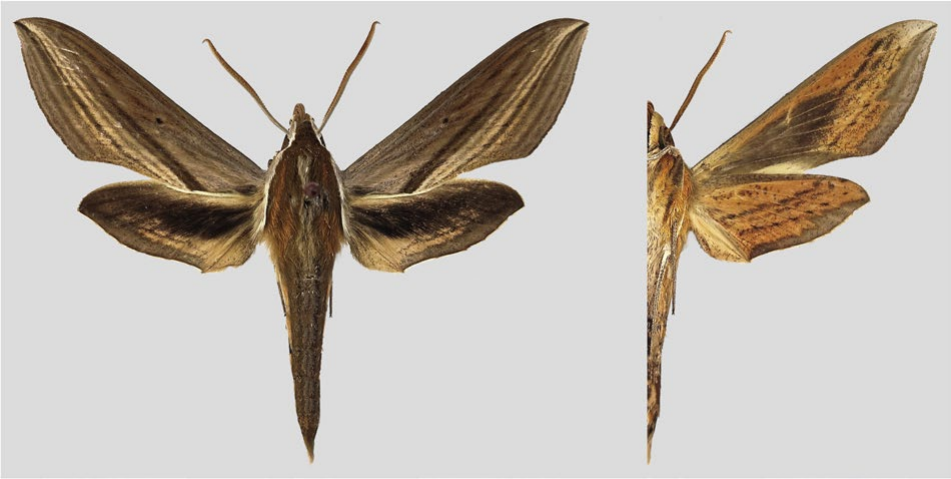
Photograph of an adult specimen of the *Theretra japonica* (Photo by Zhuo-Heng Jiang).

The adult *Theretra japonica* (Boisduval, 1869) acts as an important pollinator for a wide range of plants (Fig. 1). However, during its larval stage, it gains an unsavory reputation as a destructive pest of agricultural crops. It is a prevalent pest in Korea, Japan, Russia, and China, preferentially for damaging *Cissus, Colocasia, Hydrangea, Parthenocissus, Ampelopsis, Ipomoea batatas, Cayratia japonica* and *Vitis* (https://tpittaway.tripod.com/china/china.htm). In China, *T. japonica* can be seen almost everywhere and usually damages crops from June to October every year. Severe damage by this pest can result in complete destruction of the leaf tissue, leaving only the leaf veins and twigs, and in extreme cases, the entire plant may die. Such an infestation can significantly impair the growth and development of the affected plants.

Genomic resources containing high-quality reference genomes and transcriptomes facilitate comparisons between populations and species to answer questions ranging from broad-chromosomal evolution to the genetic basis of important adaptations^4^. A comprehensive understanding of its genome is therefore needed to promote more innovative management strategies for this destructive pest. However, genomic information of Sphingidae remains scarce. To date only 11 chromosome-level genomes have been published for species of Sphingidae (*Sphinx pinastri*, *Hyles euphorbiae*, *Hemaris fuciformis*, *Mimas tiliae*, *Laothoe populi*, *Deilephila porcellus*, *Deilephila elpenor*, *Lapara coniferarum*, *Amorpha juglandis*, *Hyles vespertilio* and *Manduca sexta*) (submission date, October 25, 2023).

Here we present a high-quality chromosome-level genome assembly of *T. japonica*, the first reference genome assembly of a member of the *Theretra*. We annotated repeated sequences, non-coding RNA (ncRNA), and protein-coding genes. This study has significant implications for the development of modern pest control strategies and serves as a reference for future genome comparison research in Lepidoptera and other insects.

## Methods

### Samples collection and sequencing

The specimens used in this study were all collected from Baishaguan Wood Inspection Station, City Shangrao, Province Jiangxi, China, on September 5, 2022. We used these five individuals for PacBio, genome survey, Hi-C and transcriptome sequencing. One male specimen was used for Hi-C, one male specimen for second-generation transcriptome sequencing, one male specimen for third-generation full-length transcriptome sequencing, and one female and one male specimen for second-generation whole genome sequencing and third-generation whole genome sequencing. We removed the intestinal tract of the samples to minimise contamination by gut microorganisms and stored the samples in liquid nitrogen at −80 °C before delivering them to the company (Berry Genomics, Beijing, China).

Third generation PacBio HiFi sequencing was performed using the SMRTbell® Express Template Prep Kit 2.0 to generate PacBio HiFi 15 K libraries. After fulfilling the quality control criteria, the DNA fragments were cut to a size of 15 Kb using the Megaruptor (Diagenode B06010001, Liege, Belgiu) instrument and concentrated using AMPure®PB Beads. The SMRTbell library construction was completed with the assistance of the 2.0 kit. Finally, fragment screening was conducted using the SageELF system. For second-generation whole-genome sequencing, the Agencourt AMPure XP-Medium kit was utilized to construct BGISEQ-500 libraries, with insert fragment sizes ranging from 200 to 400 bp. For conventional second-generation transcriptome sequencing, RNA was extracted using TRIzolTM Reagent, followed by library construction using the VAHTS mRNA-seq v2 Library Prep Kit. The Hi-C library was constructed through the following steps: crosslinking cells with formaldehyde, digesting DNA with *MboI*, filling ends and mark with biotin, ligating the resulting blunt-end fragments, purification and random shearing DNA into 300–500 bp fragments. After quality control test of the libraries using Qubit 2.0, an Agilent 2100 instrument (Agilent Technologies, CA, USA) and q-PCR, 150 bp PE sequencing of the Hi-C library were performed on the Illumina Novaseq 6000 platform by Berry Genomics Company (http://www.berrygenomics.com/. Beijing, China). Finally, we obtained 161.49 Gb of sequencing data, comprising 66.05 Gb (161.28×) of WGS data, 40.81 Gb (99.64×) of HiFi data, 34.68 Gb (84.69×) of Hi-C data, and 19.95 Gb of transcriptome data (Table 1).

**Table 1:** Sequencing data for genome assembly.

Genomic libraries	WGS	HiFi	Hi-C	RNA-sr
Sequencing data (Gbp)	66.05	40.81	34.68	9.59
Average length (bp)	150	18327.50	150	150
Sequencing coverage (x)	161.28	99.64	84.69	—

### Genome survey and assembly

The main purpose of Genome Survey analysis is to predict genome size, heterozygosity, and the proportion of repetitive sequences in order to facilitate the subsequent selection of appropriate genome assembly tools and adjustment of corresponding parameters. Firstly, the obtained second-generation BGI data obtained with fastp v 0.23.01 (‘-q 20 -D -g -x -u 10 -5 -r -c’) is subjected to quality control and trimming positions with a base quality of at least 20, removing duplicate sequences, trimming of poly-G/X tails, ensuring the proportion of disqualified bases does not exceed 10%, and correction of bases in overlapping regions^5^. The survey was derived based on the k-mer frequency distribution analysed by BBTools v38.82(https://sourceforge.net/projects/bbmap/), with the sequence length set to 21 k-mer. Genome characterization was performed using GenomeScope v2.0^6^ with the maximum k-mer coverage set to 10,000 with the parameters ‘-k 21 -p 2 -m 100000’.

High-quality HiFi reads were generated using pbccs v6.4.0, and the first rounds were assembled using the default parameters of hifiasm v0.16.1^7^ default parameters. We did not polish the assembled bases as their QV values were above 60. We used Purge_dups v1.2.5^8^ to remove redundancies in assembly based on contig similarity and sequencing depth. Minimap2 v2.4^9,10^ was used to compare HiFi reads to the genome (‘-x map-hifi’), and the genome itself (‘-xasm5 -DP’). Purge_dups was used default parameters (‘-2 -a 70’). Genome survey analysis predicts only the length of autosomal chromosomes of about 392 Mb. Second-generation sequencing was performed on female samples, with the sex chromosomes sequenced at half the depth of the autosomes, so 392 Mb should only be the length of an autosomal chromosome. The k-mer frequency distribution indicates that the genome has a low repeat content, and the potential for contamination of the data is extremely low, which can be neglected.

**Fig. 2:**
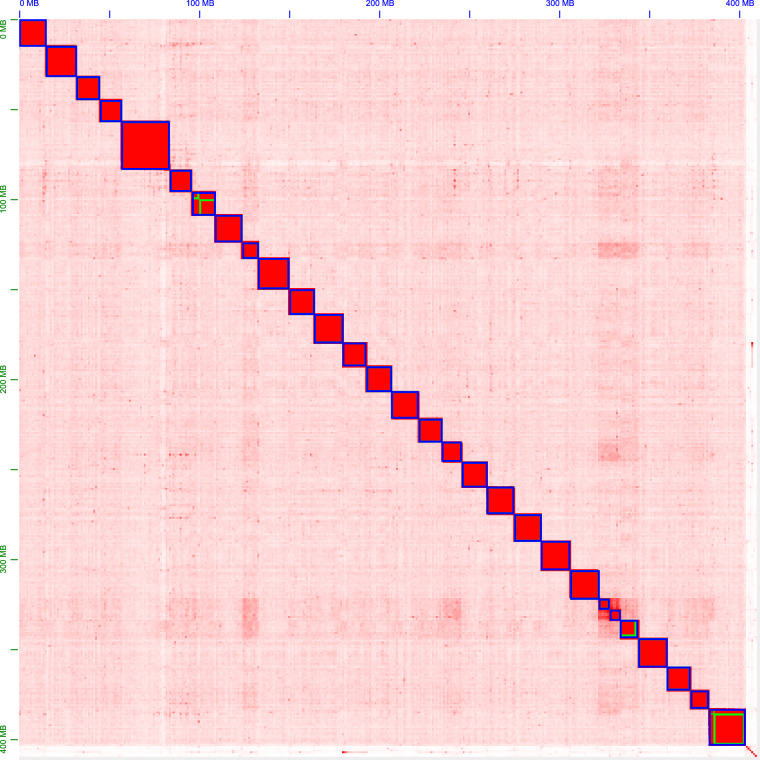
Hi-C interaction heatmaps, with each chromosome and contig framed in blue and green, respectively.

We used Hi-C data and 3D-DNA v180922^11^ for chromosome anchoring and assembly of contigs. Hi-C data were first quality controlled using Juicer v1.6.2^12^; followed by two rounds of assembly using 3D-DNA v180922. Assembly after the first round of assembly anchoring was performed using Juicebox v1.11.08^12^ for manual error correction before the second round of final anchoring. Finally, we assessed the sequencing depth of each pseudochromosome using bamtocov v.2.7.0^13^, where the input comparison bam was generated by minimap2 based on HiFi reads (‘-ax map-hifi’). The quality of chromosome assembly was extremely high, resulting in 29 chromosome-level assemblies, with only 3 chromosomes not being gap-free (Fig. 2).

**Fig. 3:**
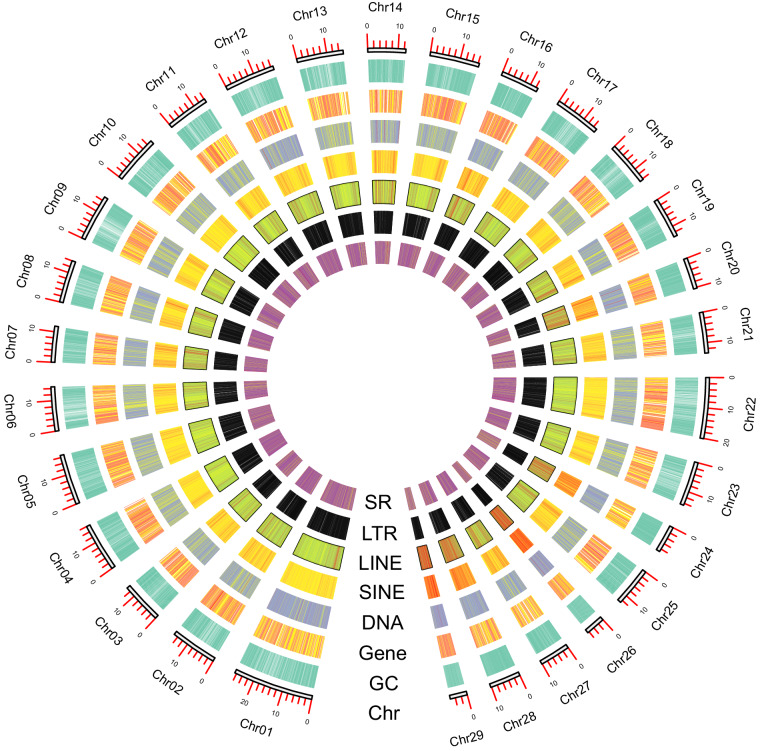
Characterization of the *Theretra japonica* genome. From the outer ring to the inner ring are the distributions of chromosome length, GC content, gene density, TEs (DNA, SINE, LINE, and LTR), and simple repeats.

Genome completeness was assessed using BUSCO v5.2.2^14^ based on the insecta_odb10 reference dataset which contains 1,367 single-copy orthologous genes. In addition, both genomic and second-generation transcriptomic raw sequences were mapped back to the genome assembly to assess the utilization of the original data and the integrity of the assembly. Minimap2 was used as the mapping tool, and mapping rates were calculated using SAMtools v1.10^15^. Possible contamination during the assembly process was investigated using MMseq2 v13^16^, performing a blastn-like search against two alignment databases: NCBI nt and UniVec. Finally, the size of the assembled genome was 409.55 Mb (Table 2 and S1), which was essentially consistent with the results of genome survey analysis. The number of scaffolds and contigs was 95 and 101 respectively, and the GC content was 37.16%. The 29 pseudochromosomes (Fig. 3) totaled 403.77 Mb, and the assembly rate was 98.59%. The genome assembly was evaluated by BUSCO, and the completeness was 99.2%, and the duplication rate was only 0.1%. The mapping ratios of second-generation survey, second-generation RNA, third-generation RNA, and third-generation Hifi data are 96.84%, 99.97%, 89.21%, and 74.43% respectively. All these indicators demonstrate that the assembly has reached an extremely high standard in terms of both continuity and completeness.

**Table 2:** Genome assembly statistics for chromosome-level assembly of *Theretra japonica*.

Genome assembly	Number
Size (bp)	409,552,430
Number of scaffolds/contigs	95/101
Number of pseudo-chromosomes (sizes)	29 (403,774,580 bp)
N50 scaffold/contig length (Mb)	14.58/14.27
GC (%)	37.16
BUSCO completeness (%)	99.2

The annotation of genomes primarily encompasses the identification and labeling of repeat sequences, non-coding RNA (ncRNA), protein-coding genes, and the delineation of gene functions. The prediction of repeat sequence was performed using RepeatMasker v4.1.2p1(http://www.repeatmasker.org) and the final database of repeat sequences was used for comparison and identification. Using RepeatModeler v2.0.3^17^ software and an additional LTR searc(‘-LTRStruct’), this de novo repeat library was created using the principle of repeat sequence specific structure and de novo prediction, which is compatible with Dfam 3.5^18^ and the RepBase −20181026^19^ database and was integrated into the final repeat sequence reference database. The results of RepeatMasker v4.1.2p1 (http://www.repeatmasker.org) and the final repetitive sequence database showed that there were 913,482 repetitive sequences (131,570,928 bp), with a percentage of repetitive sequences of 32.13%. The five categories of repetitive sequences with the highest percentage were SINE (10.77%), Unknown (8.11%), LINEs (6.85%), DNA (1.70%) and Simple Repeats (1.54%), while the percentage of LTRs was extremely low, at only 0.81%.

Two strategies were chosen for the annotation of non-coding RNA. Infernal v1.1.4^20^ was used to annotate rRNA, snRNA, and miRNA by aligning the genomic sequences with the known non-coding RNA database. For the tRNA sequences within the genome, tRNAscan-SE v2.0.9^21^ was used for prediction, and low-confidence tRNAs were filtered out using the inbuilt scripts (‘EukHighConfidenceFilter’) of the software. We obtained a total of 1,872 genomic ncRNA annotation results, including 299 rRNAs, 435 miRNAs, 117 snRNAs, 953 tRNAs, 3 ribozymes, and 2 lncRNAs. The snRNAs consisted of 83 spiceosomal RNAs (U1, U2, U4, U5, and U6), 23 C/D box snoRNAs and 5 HACA-box snoRNAs.

Predicting the structures of protein-coding genes integrate prediction results based on ab initio gene models, genes from transcriptome assembly and homologous proteins using MAKER v3.01.03^22^.

To identify the structure of protein-coding genes, we used three methods including ab initio de novo prediction of genes, comparison of transcript sequences and genomes to predict gene structures, and comparison of predictions with known protein sequences of homologous species. MAKER v3.01.03 was then used to synthesise these three types of evidence to predict the structure of protein-coding genes.

To expand the range of potential coding gene candidates by using BRAKER v2.1.6^23^ and GeMoMa v1.8^24^ and integrating both transcriptome and protein evidence, we merged the predictions of the two as an input file for MAKER *ab initio*(ab.gff3). We used MAKER to automatically train two ab initio prediction tools, Augustus v3.3.4^25^ and GeneMark-ES/ET/EP v4.68^26^, and integrated arthropod protein sequence and transcriptome data from the OrthoDB10 v1 database^27^ to improve prediction accuracy. GeMoMa(GeMoMa.c=0.4 GeMoMa.p=10) uses protein homology and intron position information to predict genes. We downloaded protein sequences from NCBI for 6 homologous species of *T. japonica* with high quality of assembly and annotation, namely *Bombyx mori* (Bombycoidea), *Drosophila melanogaster* (Diptera), *Spodoptera frugiperda* (Noctuoidea), *Pieris rapae* (Papilionoidea), *Manduca sexta* (Bombycoidea) and *Chilo suppressalis* (Pyraloidea). The transcriptome was generated using HISAT2 v2.2.0^28^ comparing the second-generation RNA-sr transcriptome data with the genome to generate BAM comparison files. We then used StringTie v2.2.0^29^ software to perform parametric assembly (‘-mix’) based on the second-and third-generation transcriptomes. Finally, we performed homology comparisons with protein sequences of homologous species downloaded from NCBI. In total, 14,614 protein-coding genes were predicted by the MAKER process, with an average gene length of 9,076.6 bp. Each gene contained an average of 7.4 exons, with an average exon length of 307.7 bp. Each gene contained an average of 6.3 introns, with an average intron length of 1146.0 bp. Each gene contained 7.2 coding sequences (CDS), and the average length was 225.4 bp. The predicted protein gene sequences were subjected to BUSCO integrity assessment, and the results were C: 99.4% [S: 69.3%, D: 30.1%], F: 0.1%, M: 0.5% (n:1367), which is higher than 99.2% of the genome score.

We have used two strategies to annotate the gene function of protein-coding genes (PCGs). The first method is to use the highly sensitive mode (‘-very-sensitive -e 1e-5’) in Diamond v2.0.11.149^30^, search the UniProtKB database for gene functions, and then compare with and the database to predict gene functions. Another method is to compare with the five comprehensive databases Pfam^31^, SMART^32^, Superfamily^33^, CDD^34^, and eggNOG v5.0^35^ to predict the conserved sequence and structural domain of proteins in gene set, Gene Ontology(GO), KEGG, Reactome, etc., the first four databases were searched InterProScan 5.53-87.0^36^, and eggNOG v5.0 database was searched with eggNOG-mapper v2.1.5^37^. Finally, the results predicted by the above two methods were integrated to obtain the final prediction of gene function. The results showed that 14,221 (97.31%) genes matched the entries in the UniProtKB database. InterProScan identified the protein structure domains in 11,890 protein-coding genes. A total of 10,425 genes were identified by InterProScan and eggNOG-mapper as GO pathway entries and 4,896 genes as KEGG pathway entries.

## Data Records

The raw sequencing data and genome assembly of *Theretra japonica* have been deposited at the National Center for Biotechnology Information (NCBI) and China National GeneBank DataBase (CNGBdb)^38^. The Hi-C, HiFi, WGS, and transcriptome data can be found under identifcation numbers SRR26855496-SRR26855499^39–42^ in NCBI and under CNP0004835 in CNGBdb. The assembled genome has been deposited in the NCBI assembly with the accession number GCA_033459515.1^43^. In addition, the annotations for repeated sequences, gene structure and functional predictions have been placed in the Figshare database^44^.

## Technical Validation

The assessment of the quality of the genome assembly has been a two-step process. Initially, we assessed the completeness of the assembly using BUSCO v5.2.2^45^ based on the insecta_odb10 database (n = 1,367). The final genome assembly displayed a BUSCO completeness of 99.2%, comprising of 99.1% single-copy BUSCOs, 0.1% duplicated BUSCOs, 0.2% fragmented BUSCOs, and 0.6% missing BUSCOs. We then calculated the mapping rate to measure assembly accuracy. The BGI, HiFi, and RNA-sr data repo rate reached 96.84%, 99.97%, and 89.21%, respectively. Overall, these assessments reflect the high quality of the genomic assembly.

### Supplementary information

appendix

## Data Availability

No specifc script was used in this work. All commands and pipelines used in data processing were executed according to the manual and protocols of the corresponding bioinformatic sofware.
