# Supplementary material for: Chromosome-level Genome Assembly of Theretra japonica (Lepidoptera: Sphingidae)
Source: Sci Data. 2024 Jul 12;11:770. doi: 10.1038/s41597-024-03500-z (PMC11245595; doi:10.1038/s41597-024-03500-z)
Supplement: Supplementary file 1 — appendix [file 41597_2024_3500_MOESM1_ESM.pdf]

TableS1. Summary of annotation of protein-coding genes of *Theretra japonica*.

**Protein-coding genes**	**Number**
Number	14,614
Mean gene length (bp)	547.3
Number of predicted protein sequences	21,307
BUSCO completeness (%)	99.4

TableS2. Summary of annotation of repeat sequences of *Theretra japonica*.

**Repetitive elements**	**Number**
Size (Mb)	131570928(32.13%)
DNA transposons (bp)	531180(0.13%)
SINEs (bp)	19518662(4.77%)
LINEs (bp)	169420(0.04%)
LTRs (bp)	9896(0.00%)
Unclassifed (bp)	33216486(8.11%)
Number of rRNA	442
Number of tRNA	1162
Number of snRNA	65
Number of scRNA	1
Number of srpRNA	4
